# Malaria in Sri Lanka: one year post-tsunami

**DOI:** 10.1186/1475-2875-5-42

**Published:** 2006-05-15

**Authors:** Olivier JT Briët, Gawrie NL Galappaththy, Priyanie H Amerasinghe, Flemming Konradsen

**Affiliations:** 1 International Water Management Institute, P.O. Box 2075, Colombo, Sri Lanka; 2 Anti Malaria Campaign Head Office, Colombo, Sri Lanka; 3 Department of International Health, University of Copenhagen, Denmark

## Abstract

One year ago, the authors of this article reported in this journal on the malaria situation in Sri Lanka prior to the tsunami that hit on 26 December 2004, and estimated the likelihood of a post-tsunami malaria outbreak to be low. Malaria incidence has decreased in 2005 as compared to 2004 in most districts, including the ones that were hit hardest by the tsunami. The malaria incidence (aggregated for the whole country) in 2005 followed the downward trend that started in 2000. However, surveillance was somewhat affected by the tsunami in some coastal areas and the actual incidence in these areas may have been higher than recorded, although there were no indications of this and it is unlikely to have affected the overall trend significantly. The focus of national and international post tsunami malaria control efforts was supply of antimalarials, distribution of impregnated mosquito nets and increased monitoring in the affected area. Internationally donated antimalarials were either redundant or did not comply with national drug policy, however, few seem to have entered circulation outside government control. Despite distribution of mosquito nets, still a large population is relatively exposed to mosquito bites due to inadequate housing. There were no indications of increased malaria vector abundance. Overall it is concluded that the tsunami has not negatively influenced the malaria situation in Sri Lanka.

## Introduction

One year ago, the authors of this article reported in this journal [1] on the malaria situation in Sri Lanka prior to the tsunami that hit on 26 December 2004, and estimated the likelihood of a post-tsunami malaria outbreak. Here they report on changes in malaria incidence recorded by the Anti Malaria Campaign (AMC) since the tsunami. Also, they discuss the control measures taken in response to the tsunami by the Anti Malaria Campaign (Additional File 1), international donors and non-governmental organizations (NGOs) (Additional File 2), and their effects on the surveillance system.

## Malaria incidence pre and post-tsunami

**Figure 1:**
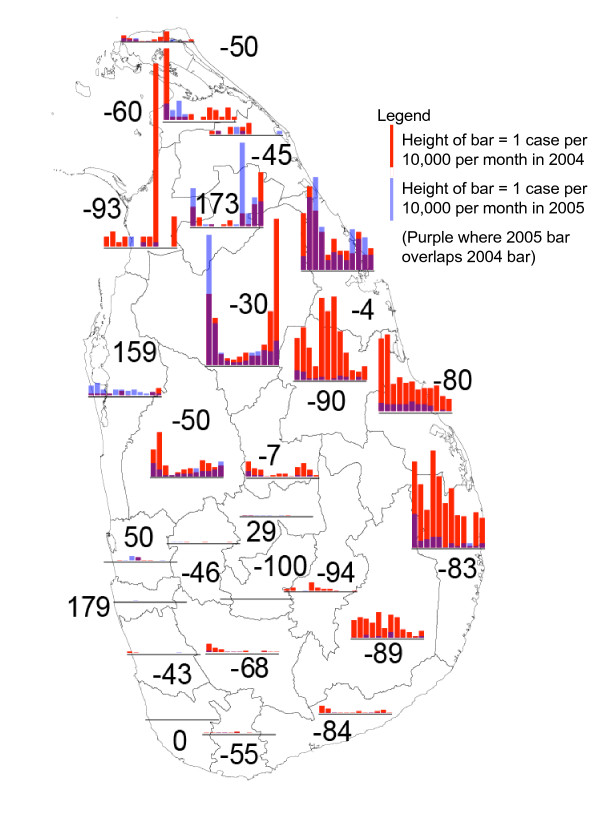
**Parasite incidence by district pre- and post-tsunami**. Monthly parasite incidence of *P. falciparum *and *P. vivax *malaria combined in 2004 (red bars) and 2005 (blue bars). Overlapping bars color purple. Bars in the legend represent 1 case per 10,000 persons per month. Numbers indicate the percentage change of January – December 2005 as compared to January – December 2004 for each district.

Figure 1 shows the monthly malaria incidence January 2004 – December 2005 at district resolution, and the percentage difference between the successive years. On the north, east and south coasts, most of the districts show a strong decline (except Trincomalee [-4%]), whereas malaria incidence increased in the districts of Colombo, Gampaha and Puttalam on the west coast, which were the least tsunami-affected coastal districts of the country. A possible reason for the slow decline of malaria in Trincomalee, when compared to other coastal districts in the east, is that, particularly in Trincomalee, there was increased civil unrest and political protests in 2005 in comparison to 2004, hindering the control efforts of the regional malaria officer (RMO). There is no indication that the physical impact of the tsunami has affected the malaria case load in Trincomalee District, as 85% of the cases were reported from Trincomalee Ministry of Health (MoH) administrative area (representing about 28% of the population in the District), which was relatively unaffected by the tsunami in terms of buildings destroyed and people killed or missing as compared to other coastal MoH administrative areas in Trincomalee district [2]. Only eighteen cases (six percent of the total cases in Trincomalee District) occurred in the Kinnyia MoH area among tsunami-displaced people. Note that, despite the "dramatic" increases in malaria incidence in some districts on the west coast, the number of cases in 2005 was low, and these increases could have arisen from incidental mini-outbreaks.

**Figure 2:**
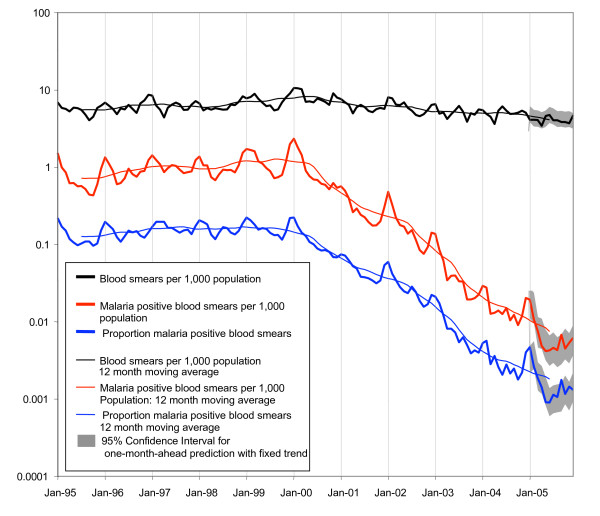
**Monthly parasite and blood smear examination incidence patterns**. Monthly parasite incidence patterns of *P. falciparum *and *P. vivax *malaria combined per 1000 population (thick red line) and 12 month moving average (thin red line), blood smears examined per 1000 population (thick black line) and 12 month moving average (thin black line), and proportion of blood smears positive for malaria (thick blue line) and 12 month moving average (thin blue line) from January 1995 to December 2005 in Sri Lanka. Ninety-five % confidence areas are indicated for one-month ahead prediction of time series for January – December 2005, using fixed trends found in the period April 2000 – December 2004 (in grey).

Although with considerable spatial variation (Table 1), the malaria incidence in Sri Lanka shows a general downward trend starting around April 2000 (Figure 2). This makes it necessary to assess whether this trend has continued after the tsunami, or whether there has been a change in trend since the disaster. A possible explanation for the downward trend pre-tsunami is the decrease in armed conflict that preceded the signing of the memorandum of understanding on the Permanent Cessation of Hostilities in February 2002. The Anti Malaria Campaign was one of the few institutions that had access to uncleared areas during the conflict, but was limited in its operation by hostilities. It should be noted that malaria transmission in Sri Lanka has always fluctuated over the years in response to major changes in control strategies and efforts, climate variations, or due to factors not yet fully established. Between April 2000 and December 2004, the malaria positive blood smear incidence in the whole country decreased exponentially by 8 % per month (the 13 month moving average [with values for months at extremes given half weight] plotted in Figure 2 is approximately a straight line on a logarithmic scale). Over the same period, the surveillance effort (the blood smears examined per population) also decreased exponentially, but only by 0.8% per month. By December 2004, the ratio of positive blood slides to the number of blood slides examined was 1:250 (400/99694). The observed monthly incidence since the tsunami, over the period January – December 2005 is not significantly different (alpha = 0.05) from the one-month-ahead prediction applying the fixed trend and using a first order auto regressive model with first order seasonal component. Therefore, at the country level, there is no evidence that the tsunami affected the incidence of malaria.

**Table 1:** Annual percentage of growth in malaria incidence over the years 2001 to 2004 as compared to the years 2004 – 2005, absolute case numbers in 2005 and population projection in districts in Sri Lanka, and their geographic position.

District	Position	Annual percent growth of incidence 2001–2004	Annual percent growth of incidence 2004–2005	Difference	Absolute cases 2005	Projected population mid 2005
Ampara	East coast	-18	-83	-65	126	612696
Anuradhapura	Inland	-50	-30	20	448	780900
Badulla	Inland	-65	-94	-30	3	829245
Batticaloa	East coast	-59	-80	-22	84	540535
Colombo	West coast	-61	179	240	17	2382298
Galle	South coast	NA*	NA*	NA*	0	1028690
Gampaha	West coast	-63	50	113	56	2112382
Hambantota	South coast	-65	-84	-18	6	536952
Jaffna	North coast	-70	-50	19	24	653466
Kalutara	West coast	-52	-43	8	4	1092711
Kandy	Inland	-59	29	88	17	1345092
Kegalle	Inland	-65	-46	19	6	798845
Kilinochchi	West coast	-89	-60	29	16	147603
Kurunegala	Inland	-61	-50	11	258	1503423
Mannar	West coast	-37	-93	-56	4	100181
Matale	Inland	-50	-77	-27	18	462498
Matara	South coast	-47	-55	-9	10	799400
Moneragala	Inland	-64	-89	-25	17	413301
Mullaitivu	East coast	-88	-43	45	5	122942
Nuwara Eliya	Inland	-55	-100	-45	0	728166
Polonnaruwa	Inland	-41	-90	-49	37	378379
Puttalam	West coast	-69	159	228	102	738475
Ratnapura	Inland	-74	-68	6	22	1068896
Trincomalee	East coast	-37	-4	33	286	355573
Vavuniya	Inland	-73	45	118	62	155650

* In Galle District, only 2 malaria cases were reported in 2001 and none thereafter. (NA = Not Available)

## Surveillance issues

Many of the tsunami-affected areas are also affected by the ethnic conflict, and this undoubtedly influences the quality of incidence records negatively in these areas. This, and spatial differences in other surveillance aspects [3] and transmission make it logical to assess the impact of the tsunami disaster and response by comparing the malaria situation in affected areas post-disaster with the situation preceding the disaster, rather than comparing affected with unaffected areas. However, surveillance was not quite the same in affected areas post-tsunami. Mobile clinics visited camps with displaced people and performed active case detection (patients with fever were encouraged to test for malaria), but results were not taken into account in the surveillance database.

Furthermore, approximately 6000 malaria rapid diagnostic kits have been supplied by UN agencies [4]. These kits are used by AMC RMOs in remote areas if microscopists are not available. Results from these tests were not included in the surveillance statistics in 2005 (but will be from January 2006 onwards). At country scale, 1000 kits per month is relatively insignificant when compared to the100,000 slides that were, on average, examined (routinely) monthly over 2005. Specifically, in tsunami affected areas, the proportion of kits relative to slides may have been higher. However, the number of cases detected but not reported by these methods (kits, mobile clinics) can be characterized as "few", although precise figures are not available (GNLG, personal communication). Overall, the surveillance effort (blood films examined) over the period January – December 2005, as per the surveillance database (Figure 2) was not significantly different from predicted. Therefore, at country level, there is no indication that the tsunami has affected surveillance capacity, neither negatively nor positively after the international aid effort.

## Tsunami medical aid and interference with country malaria treatment policy

Over 100,000 anti-malarial tablets were supplied by UN agencies after the tsunami [4]. These are chloroquine, proguanil and sulphadoxine/pyrimethamine (SP) tablets, which are in line with government policy. However, given the current low endemicity level of malaria (less than 4,000 cases were reported over the year 2004, and 1,628 cases over 2005), and the fact that government drug warehouses were well stored (50,000 tablets per district) prior to the tsunami, most of these donated tablets are likely to expire. Although stocks of some other medications have been reported "lost" from warehouses, this has not been the case for antimalarials. Some NGOs brought small quantities of artesunate-based tablets (currently not in line with government policy) into the country and were treating fever cases in camps the first week after the disaster until being asked by the AMC (RMOs visited camps when possible) to refer cases to government medical institutions or otherwise treat with government approved medication after confirmation of disease. A large shipment of unsolicited artesunate-based tablets arrived at Colombo port but was not cleared by customs after AMC orders (GNLG, personal communication).

## Vector control and personal protection since the tsunami

Six months post-tsunami, over 500,000 people were registered as tsunami-displaced [4]. of which almost 10,000 families were still staying in tents. The latest government estimates are that Sri Lanka is 21 percent of the way to its overall housing goal. So far, 7,461 new homes have been built, while homeowners have repaired another 13,737 homes. These statistics are from the government's Reconstruction and Development Agency, which is coordinating the tsunami recovery. Therefore, by government estimates, several hundred thousand Sri Lankans are still without permanent homes. Some 33,000 families, or at least 150,000 people, remain in transitional shelters. Others are living temporarily with relatives or friends [5]. Although international and national NGOs have contributed to malaria vector control by distributing over 100,000 insecticide treated nets (Additional file 2), many of the people in the camps are unable to use a mosquito net because the tents or barracks are too crowded [6], or they have difficulty placing the nets properly (particularly in tents). Also, lack of space may force displaced people to spend a lot of time outside and therefore exposed to mosquito bites. Although in tsunami-affected Aceh (Indonesia), insecticide-impregnated plastic sheeting for refugee tents and temporary houses were used, their use has not been reported in Sri Lanka. International NGOs have also contributed to malaria vector control by supplying fogging machines and helping with the development of disease awareness campaigns. After incidental reports of NGOs spraying insecticide, the government issued technical guidelines on the application of insecticides for control of vector-borne diseases in tsunami-affected areas in January 2005. In the immediate post-tsunami period, there was a programme of chlorination of wells and regular spraying of larvicides and fogging around settlement camps. Also, in some camps, RMOs have carried out indoor residual spraying. In this context, it is important to note that for *Anopheles culicifacies *and *Anopheles subpictus *A, widespread resistance to Malathion has been found and, maybe more importantly, these two mosquito species also have developed some resistance to pyrethroid insecticides now important in the operations of malaria control [7].

## Population movement

In South India, the potential migration in and out of the tsunami hit areas was initially seen as a potential risk for introducing malaria into areas with low prevalence [8]. However, a situation analysis three months after the disaster found only limited population movements in South India but highlighted the problems of increased migration into the disaster area by fishermen and pilgrims [9]. Although in Sri Lanka, (still) many people are registered as Internally Displaced Persons (IDPs) as a result of the tsunami, most of whom are hosted by friends and family or housed in camps in the vicinity of their original residences. Figures on inter-district displacement as a result of the tsunami are not available, but it can be characterized as low (International Organization for Migration Sri Lanka, UNHCR Sri Lanka, Humanitarian Information Centre for Sri Lanka, personal communications).

## Vector ecology

Densities of the principal malaria vector *An. culicifacies *and secondary vector *An. subpictus *have declined in the whole country, possibly due to a gradual switch from less effective organophosphates to pyrethroids since 2001, but it is also possible that indoor resting of vectors has been affected by the decline in use of thatched roofs (with *cadjan *or *palmyrah *leaves) with economic development; the districts in the north and east have profited less from this development. Overall, there was no indication of increased malaria vector breeding in tsunami-affected areas (R. R. Abeyasinghe, personal communication). In the tsunami-affected southern District of Hambantota, not a single *An. culicifacies *was found by the AMC entomological team in the normally high transmission month of January (2005). In a small study in tsunami-affected areas in the east coast, it was found that anophelines (*An. culicifacies *in wells, *An. subpictus *in pools after rains, *Anopheles varuna *and *Anopheles vagus *in rice fields) were breeding in all types of habitats, albeit in small numbers. Larval populations were affected by rainfall and cleaning of wells [10], as well as by chlorination and regular spraying of larvicides. On average, the coastal belt affected by salt water intrusion was about 1 km from the shore, and the salt water deposited by the tsunami dried up quickly and did not support breeding of mosquitoes. The east of the island (especially Batticaloa and Ampara Districts) received substantial rainfall within a couple of weeks after the tsunami, resulting in some flooding. This fresh water receded within a few days washing away any traces of salt water. An entomological study undertaken post-tsunami along the coast of Tamil Nadu (India) found that the urban malaria vector *Anopheles stephensi *was introduced into the area from nearby towns and became the predominant species only four weeks after the wave hit the coastline. Also in India, the rural malaria vector *An. culicifacies *seemed to adapt to higher levels of salinity than what had previously been reported, but the increasing temperatures dried up the breeding sites created in the debris left behind by the tsunami by April-May [9]. On the Andaman and Nicobar Islands, the most important vector found breeding in tsunami-affected areas was the brackish water mosquito *Anopheles sundaicus *[11]. In Sri Lanka, neither *An. sundaicus *nor *An. stephensi *is found.

## Conclusion

Despite initial warnings from some international health agencies, malaria in Sri Lanka did not increase after the tsunami. The effect of the tsunami disaster on malaria can not be accurately assessed in the presence of control measures. However, it appears that measures taken by the local health authorities in collaboration with NGOs were sufficient to prevent possible outbreaks. In addition, the ecological impact of the tsunami was not conducive to malaria vector breeding. However, still a large part of the population may be exposed to mosquito bites, despite distribution of impregnated bed nets. It is unfortunate that a large quantity of antimalarials may go to waste due to overabundant stocks, or incompatibility with the national drug policy. In emergency situations, donors and NGOs are urged to contact local health authorities for coordination.

## Authors' contributions

OJTB initiated the paper and did the analysis; GNLG supervised collection of malaria case data, and provided critical information on AMC policy; FK performed literature review; PHA supervised the mosquito breeding study in the tsunami-affected area in the east. All authors helped write, read and approved of the final manuscript.

## Supplementary Material

Additional File 1 Short/Medium Term Plan for prevention and control of possible malaria outbreaks in tsunami-affected areas of Sri Lanka. Click here for file

Additional File 2 Non-exhaustive list of reported antimalarial support by non-governmental organizations. Click here for file
