# Supplementary material for: Malaria in Sri Lanka: one year post-tsunami
Source: Malar J. 2006 May 15;5:42. doi: 10.1186/1475-2875-5-42 (PMC1475594; doi:10.1186/1475-2875-5-42)
Supplement: Additional File 1 — Short/Medium Term Plan for prevention and control of possible malaria outbreaks in tsunami-affected areas of Sri Lanka. [file 1475-2875-5-42-S1.rtf]

Short/Medium Term Plan for prevention and control of possible malaria outbreaks in tsunami affected areas of Sri Lanka. 

Prepared by the Anti Malaria Campaign Directorate on 29th December 2004.

Important: Please bear in mind that nearly all the tsunami affected districts of the country excluding the coastal districts from Matara to Gampaha belong to malarious areas. Therefore in the absence of clinical features suggestive of acute respiratory infections or acute gastro enteritis please consider the possibility of malaria in any febrile patient or in any patient with a history of fever.

Measures to be taken by Regional Malaria Officers of the Anti Malaria Campaign in tsunami affected districts and adjacent districts where camps for the displaced are located:
1.	Ensure availability of a buffer stock of at least 50000 tablets each of chloroquine and primaquine.
2.	Make available malaria diagnostic facilities (microscopy or Rapid Diagnostic Test Kits) to hospitals, camps for the displaced and to locations where outbreaks of malaria are suspected.
3.	Take immediate measures to introduce long lasting insecticide treated mosquito nets to all occupants of camps for the displaced in high risk malaria districts of Jaffna, Mullaitivu, Kilinochchi, Trincomalee, Batticaloa, Kalmunai (Ampara coastal), Hambanthota. Collect all LLINs available with GFATM partner organizations and distribute to the camps as soon as possible. Seek partner organization support for this activity if possible.
4.	Carry out indoor residual spraying of all camps for the displaced in all affected districts wit insecticide on a priority basis.
5.	Ensure that measures are taken to locate new camps for displaced persons away from malaria risk areas of the district or ensure that no malaria vector breeding sites are in close proximity to camps for displaced persons. Take measures to modify/manipulate existing breeding sites to make them unfavourable to malaria vector breeding. Consider the application of larvicides or introduction of larvivorous fish.
6.	Make arrangements with adjacent inland districts or with Anti Malaria Campaign Headquarters to have standby staff such as Public Health Inspectors, Public Health Laboratory Technicians and Public Health field Officers for deployment in tsunami-affected districts for malaria control activities.
7.	Establish communication with all foreign and local medical teams working in the area and obtain reports of suspected number of patients treated for malaria. Inform all foreign medical teams regarding National Guidelines for malaria treatment and provide copies of documents to all teams.
8.	Send fortnightly (every two weeks) a report regarding activities carried out, number of malaria cases reported, number of suspected malaria patients treated with anti malarials. Inform Director Anti Malaria regarding any outbreak of fever or malaria outbreaks immediately by telephone (Tel no. 0112588947).
