# Supplementary material for: Malaria in Sri Lanka: one year post-tsunami
Source: Malar J. 2006 May 15;5:42. doi: 10.1186/1475-2875-5-42 (PMC1475594; doi:10.1186/1475-2875-5-42)
Supplement: Additional File 2 — Non-exhaustive list of reported antimalarial support by non-governmental organizations. [file 1475-2875-5-42-S2.rtf]

Non-exhaustive list of reported antimalarial support by non-governmental organizations (source: reliefweb).

http://www.reliefweb.int/rw/rwb.nsf/db900SID/RURI-6LYPJC?OpenDocument
Source: International Federation of Red Cross And Red Crescent Societies (IFRC)
Date: 13 Feb 2006
South Asia: Earthquake & Tsunamis Third/Fourth Quarterly Report Appeal No.28/2004 Operation Update No. 58
Distribution of mosquito nets in Ampara district (with ministry of health) together with appropriate health education

http://www.reliefweb.int/rw/rwb.nsf/db900SID/EVOD-6LUECU?OpenDocument
Source: Humanitarian Situation Report - Sri Lanka: 03 - 09 Feb 2006
United Nations Office for the Coordination of Humanitarian Affairs (OCHA)
Date: 09 Feb 2006
IOM has distributed over 700 bags of rice, 500 hurricane lamps, 300 packets of tea, 15 tents, and 37 mosquito nets to families displaced from transitional shelter sites in Trincomalee district due to the prevalent political situation

http://www.reliefweb.int/rw/rwb.nsf/db900SID/KHII-6LJ8PE?OpenDocument
Source: United Nations Office for the Coordination of Humanitarian Affairs (OCHA)
Date: 26 Jan 2006
Humanitarian Situation Report - Sri Lanka: 21 - 26 Jan 2006
“A total of 744 bed-nets and 50 baby kits in Valachenai, Batticaloa in collaboration with Regional Malaria Officer.”

http://www.reliefweb.int/rw/rwb.nsf/db900SID/KHII-6KWA69?OpenDocument
Source: MADRE (Madre)
Date: 10 Jan 2006
The tsunami: One year later
Supplies for pregnant women and new mothers, including infant mosquito nets and mats

http://www.reliefweb.int/rw/rwb.nsf/db900SID/ETOA-6KW3CX?OpenDocument
Source: The Salvation Army
Date: 30 Nov 2005
East Asian Tsunami Recovery Report - Nov 2005
Sector: Nutrition, health or medical services; Objectives: Provide medical exams/referral, establish health clinics, distribute mosquito nets; Beneficiaries to Date: 1,502.

http://www.reliefweb.int/rw/rwb.nsf/db900SID/RMOI-6KH3JL?OpenDocument
Source: GOAL
Date: 23 Dec 2005
What GOAL has achieved in Sri Lanka after the tsunami one year on
distributing mosquito nets

http://www.reliefweb.int/rw/rwb.nsf/db900SID/RMOI-6KG5HB?OpenDocument
Source: Concern
Date: 26 Dec 2005
Concern Sri Lanka - One year on
Project Galle 2005 was established in response to the tsunami by a group of Sri Lankan and international volunteers living in the Galle district. The group identified families and distributed 7,376 family kits including essential relief items. Family kits included a mosquito net, a sleeping mat, water collection vessel, and essential cooking and eating utensils. Personal hygiene items were also added. 

http://www.reliefweb.int/rw/rwb.nsf/db900SID/KHII-6KK5CS?OpenDocument
Source: United Nations Children's Fund (UNICEF)
Date: 22 Dec 2005
UNICEF Situation Report Sri Lanka 27 Nov - 22 Dec 2005
In Kilinochchi, assistance to people affected by the October floods in Kilinochchi and Mullaitivu has continued during the reporting period. UNICEF responded by distributing tarpaulins, 1000 liter water tanks, water pumps, sleeping mats, mosquito nets

http://www.reliefweb.int/rw/rwb.nsf/db900SID/KHII-6KG4GG?OpenDocument
Source: International Federation of Red Cross And Red Crescent Societies (IFRC)
Date: 15 Dec 2005
Tsunami operation - Facts and figures updated 15 Dec 2005
More than 300,000 people have received distributions of relief goods including food, cooking supplies, stoves, hygiene items, mosquito nets, lamps, clothes, sleeping mats, school uniforms, schoolbags, stationery and clothes.

http://www.reliefweb.int/rw/rwb.nsf/db900SID/RMOI-6K53TS?OpenDocument
Source: Direct Relief International
Date: 15 Dec 2005
Tsunami grant summaries
The Tropical and Environmental Diseases and Health Association (TEDHA) was founded in Hikkaduwa, Sri Lanka to address environmental concerns in the country. Comprised of environmental health specialists, an epidemiologist, parasitologists, and public health inspectors, the organization has worked closely with the Ministry of Health on a national malaria control program. Following the tsunami, TEDHA initiated voluntary tsunami relief health activities in Thotagamuwa-Hikkaduwa, including a vector control assessment of the area. This assessment confirmed a strong need for vector control measures in the area, especially in relief camps. 
With a grant from Direct Relief, TEDHA has implemented vector borne disease prevention programs in Hikkaduwa and Hambantota, both located on the southern coast of Sri Lanka, and both devastated by the tsunami. Hikkaduwa was severely affected by the tsunami, suffering approximately half of the Galle District's nearly 5,000 deaths. Hambantota District sustained over 5,000 causalities making it one of the worst affected districts. For survivors, a lack of housing, an unsafe water supply, limited nutritional supply, and poor hygiene conditions contribute to an increased risk of communicable diseases such as diarrhea, dengue, malaria, filariasis, and Japanese encephalitis. 
In Hikkaduwa and Hambantota, TEDHA has distributed 18,000 insecticide treated mosquito nets, procured by Direct Relief, to families residing in displaced persons camps and affected neighborhoods. Recipients receive training on use of the nets and on measures to effectively prevent disease. Bed nets will be retreated as needed by TEDHA's community health volunteers who will regularly monitor net use.
In addition, Direct Relief provided 5,000 insecticide-treated mosquito nets, with a value of $30,457, to Sarvodaya to assist in their (separate) vector control programs in relief camps.

http://www.reliefweb.int/rw/rwb.nsf/db900SID/RMOI-6K53H7?OpenDocument
Source: Direct Relief International
Date: 15 Dec 2005
One year after tsunami, Direct Relief International remains committed to providing vital medical resources
Over 168,800 families in Sri Lanka and India are being protected from malaria and other insect-borne diseases through the provision of 170,100 insecticide-treated mosquito nets and fogging devices;

http://www.reliefweb.int/rw/rwb.nsf/db900SID/KHII-6K375J?OpenDocument
Source: Church World Service (CWS)
Date: 14 Dec 2005
Tsunami anniversary: In global agency¹s largest natural disaster response, aid workers see signs of recovery at people level
In Sri Lanka, Church World Service¹s Pakistan-Afghanistan regional staff provided food and water, tents, mats, sheets, mosquito nets, health supplies, kitchen utensils, clothing and medicine to some 56,100 families.

http://www.reliefweb.int/rw/rwb.nsf/db900SID/KOCA-6JZJ9G?OpenDocument
Source: Medical Emergency Relief International (Merlin)
Date: 12 Dec 2005
Preventing disease outbreaks 
Thousands of people have been living in makeshift camps which are overcrowded and mosquito-ridden. In these difficult conditions, maintaining standards of personal hygiene and cleanliness are paramount if people are to stay healthy. Merlin has been helping to prevent disease outbreaks in camps by distributing more than 15,000 hygiene kits, organising clean-ups and providing camp cleaning equipment. Merlin has also trained more than 800 volunteers to promote good hygiene practices in camps. 
Merlin's project is reaching more than 1 million people in total, including 3,000 mothers who have been given essential items for their newborn babies, and 120,000 people who have received mosquito nets. Two hundred midwives have also been provided with emergency delivery equipment and supplies from Merlin. 
Over the coming months, Merlin will construct and equip seven permanent health centres to replace those that were destroyed. These facilities together served more than 100,000 people before the tsunami. Merlin is also helping to strengthen the existing health systems, for example, by training health workers, improving laboratories and helping to develop an emergency operational plan for disease outbreaks.

http://www.reliefweb.int/rw/rwb.nsf/db900SID/DPAS-6JPF3V?OpenDocument
Source: Adventist Development and Relief Agency International (ADRA)
Date: 01 Dec 2005
South Asia: ADRA publishes report on tsunami response and launches remembrance campaign
Provision of mosquito nets.

http://www.reliefweb.int/rw/rwb.nsf/db900SID/RMOI-6K88CA?OpenDocument
Source: Swiss Agency for Development and Cooperation (SDC)
Date: 30 Nov 2005
Two projects in Sri Lanka: Reconstruction of schools and houses
The first SDC/HA measure was the shipment of 150 truckloads of locally acquired relief goods (including mats, covers, mosquito nets, water cans, cooking sets, soap, toiletries, candles, matches) for around 3,000 homeless families in Matara district. 250 water tanks were organized and set up to ensure a supply of drinking water in Matara.

http://www.reliefweb.int/rw/rwb.nsf/db900SID/RMOI-6KS3AJ?OpenDocument
Source: Government of Canada
Date: 24 Nov 2005
12,000 mosquito nets and floor mats were distributed

http://www.reliefweb.int/rw/rwb.nsf/db900SID/RMOI-6HH76M?OpenDocument
Source: United Nations Office for the Coordination of Humanitarian Affairs (OCHA)
Date: 21 Oct 2005
Humanitarian Situation Report - Sri Lanka: 15 - 21 Oct 2005
Dengue alerts are ongoing in Matara district and among aid workers the OCHA Galle field office reports. Two cases of dengue have been reported by the Spanish Red Cross and Caritas International. IOM Sri Lanka is in the process of developing an information campaign aimed at delivering messages about the high tendency for an outbreak of dengue and malaria due to increased breeding sites following the seasonal rains. In Trincomalee, the Medical Health Officer with the support of IOM launched an awareness raising campaign on dengue and malaria prevention, which will be aired through the local cable TV network. This announcement will be telecast in local languages, initially for seven continuous days and then every Sunday through December 2005. 
The broadcast message states: "There is potential risk for outbreaks of dengue and malaria in Trincomalee again this season. Let us protect ourselves from these diseases by destroying mosquito breeding sites such as bottles, coconut shells, polythene bags, plastic containers and other places where water can stagnate." 
In Batticaloa, as part of the prevention exercise for dengue and malaria, an environmental hygiene programme has been developed by the CHSO (community health surveillance officer) to inform residents on how to destroy breeding sites for vectors of the diseases. 

http://www.reliefweb.int/rw/rwb.nsf/db900SID/KHII-6GQ7ZY?OpenDocument&cc=lka
Source: United Nations Office for the Coordination of Humanitarian Affairs (OCHA)
Date: 29 Sep 2005
Humanitarian Situation Report - Sri Lanka: 23 - 29 Sep 2005
In Trincomalee, an anti-malaria campaign and vector surveillance was recently conducted by by health officials in Kinniya and Trincomalee town. Four malaria cases have been reported from Kinniya. UNICEF distributed 1,000 insecticide treated nets in Kinniya. Furthermore, a dengue fever awareness programme is being conducted by 100 community volunteers trained by the Ministry of Health, every Saturday in Trincomalee. Public warning messages are being issued to prevent an outbreak of dengue fever. 

http://www.reliefweb.int/rw/rwb.nsf/db900SID/RMOI-6EUAW7?OpenDocument
Source: United Nations Children's Fund (UNICEF)
Date: 28 Jun 2005
UNICEF Situation Report Sri Lanka 28 Jun 2005
66,000 families served with kits including hygiene materials, mosquito nets, lanterns, cooking utensils, buckets, water purification tablets, clothes, mattresses and sleeping bags.

http://www.reliefweb.int/rw/rwb.nsf/db900SID/KHII-6DY5LD?OpenDocument
Source: United Nations Office for the Coordination of Humanitarian Affairs (OCHA)
Date: 16 Jun 2005
Sri Lanka: Facts regarding post-tsunami recovery six months on
- 101 emergency health kits have been provided to hospitals and clinics by UNICEF and WHO benefiting some 1,500,000 tsunami-affected people
- Approx. 6000 malaria rapid diagnostic kits and over 100,000 anti-malarial tablets supplied by UN agencies.
- 48,000 impregnated mosquito nets have been provided by UNICEF and WHO with 50,000 more are on the way.

http://www.reliefweb.int/rw/rwb.nsf/db900SID/EVOD-6CYHRE?OpenDocument&cc=lka
Source: United Nations Office for the Coordination of Humanitarian Affairs (OCHA)
Date: 16 Jun 2005
Sri Lanka: Facts regarding post-tsunami recovery six months on
Five-thousand mosquito nets were handed over to the Deputy Director of Health Services in Ampara by UNICEF to support the Anti-Malaria Campaign. Another 5,400 nets were provided to the Health Sevice in Jaffna district. An increase in mosquito-borne diseases is likely due to the seasonal North East monsoon rains.

http://www.reliefweb.int/rw/rwb.nsf/db900SID/RMOI-6DV3DH?OpenDocument
Source: Lutheran World Relief (LWR)
Date: 30 Jun 2005
Six months later: a tsunami update from Lutheran World Relief
Provision of mosquito nets.

http://www.reliefweb.int/rw/rwb.nsf/db900SID/SODA-6DS8J9?OpenDocument
Source: Medair
Date: 27 Jun 2005
Tsunami crisis: 6 month review of Medair activities in Sri Lanka
Essential relief items distributed to 2,300 families including; buckets, jerry cans, mosquito nets, mats, milk powder, soap, sachets of water purification chemicals.

http://www.reliefweb.int/rw/rwb.nsf/db900SID/EVOD-6DCGME?OpenDocument
Source: United Nations Office for the Coordination of Humanitarian Affairs (OCHA)
Date: 14 Jun 2005
IOM/OCHA Sri Lanka: Humanitarian Situation Report 10 - 14 Jun 2005
The situation in Mandana camp, Thirukkovil division, Ampara district provides a good example of how the government, UN agencies and NGOs remain pro-active in their response to continuing humanitarian relief concerns. As recently as 9 June, the population of the Mandana camp, which held 592 families in March, was down to 161 families. Construction of transitional shelters, lack of transportation and incidents of violence had caused some residents to relocate, but the principal reason for the exodus was a report of several cases of Hepatitis A which quickly evolved into a rumour of the existence of a yellow fever outbreak, prompting a camp-wide scare. UN agency and NGO representatives with government health authorities took swift preventive measures, including safeguarding water supplies, spraying for mosquitoes and stepping up awareness raising activities regarding Hepatitis A and a campaign to assure residents there was no evidence of yellow fever. 

http://www.reliefweb.int/rw/rwb.nsf/db900SID/EVOD-6CYHRE?OpenDocument
Source: United Nations Office for the Coordination of Humanitarian Affairs (OCHA)
Date: 02 Jun 2005
Humanitarian Situation Report - Sri Lanka: 27 May - 2 June 2005
Five-thousand mosquito nets were handed over to the Deputy Director of Health Services in Ampara by UNICEF to support the Anti-Malaria Campaign. Another 5,400 nets were provided to the Health Sevice in Jaffna district. An increase in mosquito-borne diseases is likely due to the seasonal North East monsoon rains. 

http://www.reliefweb.int/rw/rwb.nsf/db900SID/EVOD-6CPHQ5?OpenDocument
Source: United Nations Office for the Coordination of Humanitarian Affairs (OCHA)
Date: 24 May 2005
IOM/OCHA Sri Lanka: Humanitarian Situation Report 20 - 24 May 2005
WHO is providing fogging machines for mosquito control against malaria and dengue to a number of Deputy Provincial Directors of Health (DPDH) Services in the districts. In addition, WHO has recently conducted a fogging machine operation and maintenance workshop in Matara district for new operators of the fogging equipment and for Public Health Inspectors from Galle, Matara and Hambantota districts.

http://www.reliefweb.int/rw/rwb.nsf/db900SID/VBOL-6CJHBG?OpenDocument
Source: United Nations Office for the Coordination of Humanitarian Affairs (OCHA)
Date: 19 May 2005
Humanitarian Situation Report - Sri Lanka: 13 - 19 May 2005
Some 12,400 mosquito nets, which were handed over by UNICEF to the Deputy Director of Health Services in Batticaloa district, are being distributed to families in the malaria prevalent areas of the district. An increase in Mosquito-borne diseases is likely due to the seasonal North East monsoon rains.

http://www.reliefweb.int/rw/rwb.nsf/db900SID/LSGZ-6CNBNN?OpenDocument
Source: Oxfam
Date: 04 May 2005
Tsunami crisis - situation update: Providing shelter, rebuilding livelihoods
In Trincomalee, a temporary mobile shed for public performance was made and erected in Narasima Malai camp. In Kuncahavalli a drama on diarrhoea, hygiene and mosquito borne diseases was performed to 100 people living in Narasima Mali camp and 75 people from Alnuriya Vidiyalayam camp. 

http://www.reliefweb.int/rw/rwb.nsf/db900SID/MHII-6BZ5MX?OpenDocument
Source: American Red Cross
Date: 27 Apr 2005
Red Cross spurs anti-malaria campaign
By Alice Kociejowski and Stacey M. Winston, special to Redcross.org 
Wednesday, April 27, 2005 -- Colombo, Sri Lanka -- It has been four months since the tsunami, and the International Red Cross/Red Crescent Movement continues to provide invaluable assistance to vulnerable families -- notably it began an anti-malaria campaign. 
At the request of the Ministry of Health, the Sri Lankan Red Cross (SLRCS) and the International Federation of Red Cross and Red Crescent Societies (Federation), in cooperation with the International Committee of Red Cross (ICRC), are distributing treated mosquito nets across the Ampara district to tsunami affected and indirectly affected families, as part of a nationwide anti-malaria campaign. 
Ampara has one of the highest incidence rates of malaria in Sri Lanka, and mosquito nets, if used properly, provide simple but effective prevention against this deadly disease. 
"Malaria can be a difficult disease to control, especially in a post-disaster situation, but mosquito nets are one of the easiest and most effective solutions," said Jeff Chinn, American Red Cross relief team member. 
As the rainy season approaches and the risk of vector borne diseases increases, distribution of mosquito nets becomes more essential. Since the tsunami struck, the SLRCS and the Federation have distributed over 66,000 nets in Galle, Matara, Hambantota and Ampara. 
This distribution is just a small, but vital part of the Red Cross work that aims to improve the lives of vulnerable people in all areas of Sri Lanka. 

http://www.reliefweb.int/rw/rwb.nsf/db900SID/KHII-6JF83M?OpenDocument
Source: International Committee of the Red Cross (ICRC)
Date: 04 Apr 2005
Tsunami disaster in Sri Lanka : The response of the International Committee of the Red Cross (ICRC) 4 Apr 2005
Working with the local authorities, the Sri Lankan Red Cross and the ICRC have delivered over 35,000 family kits to welfare centres and transit camps and other displaced persons in the north and east of the country. Such kits typically contain floor mats, bed sheets, soap, towels, buckets, jerry cans and plastic dishes. In addition, over 100 welfare centres in the same regions were provided with cooking pots and utensils for communal cooking. Nearly 12,000 pieces of clothing, 22,000 blankets, 11,000 kitchen sets and 3,300 kerosene lamps have been distributed to displaced families. The ICRC is assisting the Canadian, Japanese, Swiss, Austrian and American Red Cross Societies to provide 30,000 displaced families with monthly hygiene kits over a six-month period. The kits contain soap, toothpaste, sanitary towels, bath towels, mosquito coils, etc.

http://www.reliefweb.int/rw/rwb.nsf/db900SID/KHII-6B53VW?OpenDocument
Source: United Nations Office for the Coordination of Humanitarian Affairs (OCHA)
Date: 02 Apr 2005
Indonesia, Sri Lanka, Thailand: Earthquake and Tsunami OCHA Situation Report No. 34
In Ampara, the local health service is coordinating the distribution of 70,618 insecticide-treated mosquito nets supplied by several agencies. The total mosquito net requirement for the district is 83,802.

http://www.reliefweb.int/rw/rwb.nsf/db900SID/VBOL-6AZHDG?OpenDocument
Source: United Nations Office for the Coordination of Humanitarian Affairs (OCHA)
Date: 31 Mar 2005
Humanitarian Situation Report - Sri Lanka: 25 - 31 Mar 2005
In Ampara, the local health service is coordinating the distribution of insecticide-treated mosquito nets by a number of agencies including: UNICEF, 20,000 nets, GOAL, 20,000, Merlin, 2,450, MSF, 3,900, ICRC, 5,000, LIONS, 15,000 and Medair 4,268. The total mosquito net requirement for the district is 83,802.

http://www.reliefweb.int/rw/rwb.nsf/db900SID/RMOI-6AS2SF?OpenDocument
Source: Caritas
Date: 24 Mar 2005
Caritas reviews Sri Lanka programmes - three months after tsunami
In Galle division mosquito nets have also been distributed to families in temporary shelters as malaria and dengue fever are endemic to the coastal areas of Sri Lanka.

http://www.reliefweb.int/rw/rwb.nsf/db900SID/EGUA-6ARQF6?OpenDocument
Source: Oxfam
Date: 23 Mar 2005
OI Tsunami External Bulletin #24 of 23 Mar 2005
In Matara and Hambantota, a distribution of non-food-relief items was successfully completed, and local women and men received 10,834 hygiene items, including nappies, hot water flasks, soap, mosquito nets, washing bowls, feeding cups and spoons, a cleaning brush for bottles, sponge, sheets and a pillow. Family packs were also distributed.

http://www.reliefweb.int/rw/rwb.nsf/db900SID/VBOL-6AQJW6?OpenDocument
Source: International Organization for Migration (IOM)
Date: 21 Mar 2005
IOM Sri Lanka: Tsunami response program update 21 Mar 2005
IOM distributed 326 mosquito nets in two IDP camps in Eachchilampattu DS division on behalf of Trincomalee Lions Club.

http://www.reliefweb.int/rw/rwb.nsf/db900SID/RMOI-6AL8UV?OpenDocument
Source: Medair
Date: 17 Mar 2005
South Asia Emergency - Operations update
Distribution of essential relief items to 2,294 families including; 2,247 buckets, 2,161 jerry cans, 4,258 mosquito nets, 2,114 mats, 2,088 boxes of milk powder, 1070 bars of soap, 77,580 sachets of water purification chemicals.

http://www.reliefweb.int/rw/rwb.nsf/db900SID/RMOI-6AL4PE?OpenDocument
Source: HelpAge International
Date: 17 Mar 2005
After the tsunami: Latest from Sri Lanka
HelpAge Sri Lanka is working with four local community organisations in Ampara, Baticaloa and Trincomalee districts to distribute non-food items such as pots, pans, mosquito nets, soap, disinfectants, spoons, knives, plates, and other items required to cope with daily needs. 

http://www.reliefweb.int/rw/rwb.nsf/db900SID/VBOL-6B8ERY?OpenDocument
Source: ZOA Refugee Care
Date: 08 Feb 2005
Update on ZOA tsunami relief work in Sri Lanka - 8 Feb 2005
Dispatching of the first of 90,000 high quality mosquito nets commenced (distribution to be completed before the end of June).
